# Polymerization of Various Lignins via Immobilized *Myceliophthora thermophila* Laccase (MtL)

**DOI:** 10.3390/polym8080280

**Published:** 2016-08-03

**Authors:** Daniela Huber, Alessandro Pellis, Andreas Daxbacher, Gibson S. Nyanhongo, Georg M. Guebitz

**Affiliations:** 1 Institute of Environmental Biotechnology, University of Natural Resources and Life Sciences, Vienna, Konrad Lorenz Str. 20, 3430 Tulln, Austria; daniela.huber@boku.ac.at (D.H.); andreas.daxbacher@students.boku.ac.at (A.D.); g.nyanhongo@boku.ac.at (G.S.N.); guebitz@boku.ac.at (G.M.G.); 2 Department of Biology, Botswana International University of Science and Technology, Private Bag 16, Plot 10017 Palapye, Botswana; 3 Austrian Centre of Industrial Biotechnology ACIB GmbH, Konrad Lorenz Str. 20, 3430 Tulln, Austria

**Keywords:** lignin, biocatalyzed polymerization, enzyme immobilization, laccase, green processing

## Abstract

Enzymatic polymerization of lignin is an environmentally-friendly and sustainable method that is investigated for its potential in opening-up new applications of one of the most abundant biopolymers on our planet. In this work, the laccase from *Myceliophthora thermophila* was successfully immobilized onto Accurel MP1000 beads (67% of protein bound to the polymeric carrier) and the biocatalyzed oxidation of Kraft lignin (KL) and lignosulfonate (LS) were carried out. Fluorescence intensity determination, phenol content analysis and size exclusion chromatography were performed in order to elucidate the extent of the polymerization reaction. The collected results show an 8.5-fold decrease of the LS samples’ fluorescence intensity after laccase-mediated oxidation and a 12-fold increase of the weight average molecular weight was obtained.

## 1. Introduction

Lignin is, besides cellulose and hemicellulose, one of the major components of lignocellulosic biomass and one of the most abundant polymers in nature. It is a complex and highly cross-linked amorphous copolymer synthesized from random polymerization of three primary phenylpropane monomers, namely para-coumaryl alcohol, coniferyl alcohol, and sinapyl alcohol [1]. The chemical structure of lignins produced by the pulp and paper industry depends on the plant source and on the pulping process [2]. The two most common processes for lignin extraction from wood are the Kraft and the sulfite processes. In the Kraft process, lignin is highly fragmented and it is mainly burnt for energy production [3]. The lignin derived from the sulfite process has a higher molecular weight and water-soluble lignosulfonates are obtained, which makes this material interesting as technical surfactant, plasticizer with dispersing abilities, in the oil industry, etc. [3,4]. Since lignin is a cheap and underutilized resource, there is an increasing interest in upgrading this carbon-rich hetero-polymer. When derived from the pulp and paper industry, lignin is a heterogeneous mixture with a broad molecular weight distribution, which has an impact on the polymer properties or flow behavior [2]. For example, when chemical pre-treated lignosulfonate was added to polymer blends, a deterioration of the mechanical properties due to the poor adhesion and dispersion of lignin particles was observed [5]. Therefore, an increase in molecular weight could improve the dispersion properties, which are important for the usage as plasticizer or dispersant.

Enzymatic polymerization is a sustainable and environmentally friendly option to increase the molecular weight of lignin [3]. Enzymes that are involved in the natural lignification process like peroxidases or laccases are a good choice as biocatalysts for such polymerizations [6]. In this work, laccases (EC 1.10.3.2) (Enzyme Commission Number) were investigated for their ability of polymerizing various lignins since these multi-copper oxidases are known to catalyze the mono-electronic oxidation of substrates (phenols, aromatic, or aliphatic amines) to the corresponding reactive radical, which can lead to the formation of dimers, oligomers, and polymers [7]. It is well-known that laccase-mediated polymerization may cause condensation reactions between the phenoxy radicals that are formed resulting in new C–C, aryl-aryl, ether, or aryl-alkyl cross-linkages [5]. Ortner et al. studied the oxidation of various industrial lignins (Organosolv, Indulin and lignosulfonates) aqueous solutions and the potential of external oxygen supply was demonstrated, which was also strengthened by recent findings [8,9]. Additionally, polymerization of various lignins (Organosolv hardwood lignin, soda wheat straw lignin, alkali pretreated wheat straw lignin, kraft softwood, etc.) was performed in organic media using similar biocatalysts [10]. A study by Areskogh et al. investigated the polymerization of unmodified lignosulfonate salts with two different enzymes to improve the molecular weight for the usage as plasticizers [3]. Hence, several studies concerning the free enzyme based polymerization of lignins were already reported, but at the best of our knowledge, the usage of immobilized laccases for lignin polymerization was not yet exploited.

Compared with free enzymes, immobilized enzymes present several advantages such as recyclability, improved pH and thermal stability, easy separation from the reaction mixture and possibility to be used in flow and continuous reactors [10,11]. Several immobilization techniques on polymer supports are already available [10,11,12,13] and various parameters (e.g., glycosylation, size of the enzyme, isoelectric point, carrier material) have to be considered [14,15]. In this study, laccases were immobilized on Accurel MP1000 beads (3 M Deutschland GmbH, Wuppertal, Germany) for the polymerization of various lignin substrates was carried out.

## 2. Materials and Methods

### 2.1. Materials

Laccase from *Myceliophthora thermophila* (MtL) was purchased from Novozyme (Bagsværd, Denmark). Magnesium(Mg)-lignosulfonate (LS) was derived from the evaporation plant with a dry content of 30%. Polypropylene beads (Accurel MP1000 surface area of 55.985 m^2^·g^−1^, particle density of 1.993 g·cm^−3^ and particle diameter <1500 mm) were purchased from 3 M Deutschland GmbH (Wuppertal, Germany). EC–EP/M Sepabeads (average pore diameter 10–20 nm, particle size range 200–500 μm, water retention 55%–65%) were kindly donated by Resindion S.R.L., (Mitsubishi Chemical Corporation, Milan, Italy). Folin Cioulteau (FC) reagent, methoxyethanol, poly(styrenesulfonic acid sodium salt), Kraft lignin and all other chemicals were purchased in analytical grade by Sigma-Aldrich (St. Louis, MO, USA) and used as received if not otherwise specified.

### 2.2. Enzyme Activity

Laccase activity was determined according to Prasetyo et al. [5] with some modifications. Briefly, oxidation of ABTS (2,2’-Azino-di-(3-ethylbenzthiazolin-6-sulfonsäure)) in 50 mM sodium phosphate NaPi buffer pH 7 to its cation radical was monitored at 420 nm using a Tecan Infinite 200 Pro spectrophotometer (Tecan, Zürich, Switzerland). The activity was calculated in katal (kat) and corresponds to the amount of laccase converting 1 mole of ABTS per second.

### 2.3. Protein Concentration

The protein concentration was determined with the bicinchoninic acid (BCA) method (Sigma-Aldrich, St. Louis, MO, USA). The BCA kit was diluted 5× in mQH_2_O and 10 µL of the sample were incubated with 200 µL of BCA reagent for 5 min at 400 rpm. Absorbance was measured with a Tecan Infinite 200 Pro spectrophotometer (Tecan, Zürich, Switzerland) at 595 nm. The protein concentration was calculated by means of a bovine serum albumin standard curve.

### 2.4. Immobilization of Laccases on Polymeric Supports

Polypropylene (PP) beads were washed three times in ethanol under vacuum and three times with mQH_2_O. 1.5 g of PP beads were dispersed in the immobilization buffer (50 mM NaPi buffer pH 7) and laccase was added to a final protein concentration of 1% w·w^−1^ based on the total amount of PP beads. The immobilization process was monitored by taking samples hourly between 0 and 8 h, and after 24 h. The enzyme activity and the protein concentration of the supernatant were determined as described above. Immobilization on EC–EP beads was performed following a previously reported protocol using NaPi buffer as immobilization media. The unreacted epoxy groups were blocked incubating the preparation in a glycine solution for 24 h as previously described [16]. All immobilized preparations were washed three times with 50 mM NaPi buffer pH 7 and dried under vacuum for 48 h prior to use. The obtained preparations will further be called MtL–PP and MtL–EP.

### 2.5. Polymerization of Lignin with Immobilized Enzyme

First, 5 mL of a 10% w·v^−1^ solution of Kraft lignin (KL) or lignosulfonate (LS) were prepared in double distilled water (ddH_2_O) and the pH was adjusted to 7, which is the optimal pH for the used laccase [8]. In addition, 50 or 100 mg of immobilized preparation were then added to the reaction solution and the polymerization process was conducted for 24 h at 21 °C and 400 rpm. Samples were taken hourly between 0 and 8 h, and after 24 h of the reaction and the phenol content, fluorescence intensity and molecular weight were analyzed. Additionally, the same reaction set-up with O_2_ supply was investigated. Pure oxygen was blown in excess with a flow rate of 10 cm^3^·min^−1^ during the whole polymerization process. Monitoring was performed as previously described.

The oxygen levels were monitored using a Firesting device (Pyroscience GmbH, Aachen, Germany). The device consists of an immobilized indicator dye in a glass reaction vessel. The oxygen saturation of the reaction solution was measured by an excitation of 620 nm and at an emission of 760 nm by quenching the luminescence of the oxygen indicator before calibrating the system to 100% oxygen with pure oxygen and 0% oxygen with nitrogen [17].

### 2.6. Fluorescence Intensity and Phenol Content Measurements

The decrease in fluorescence intensity was measured over the whole polymerization process as previously described [8]. In addition, 100 µL of lignin solution were incubated with 120 µL of a methoxyethanol:water (2:1 v·v^−1^) solution. The fluorescence intensity was measured at an excitation of 355 nm and an emission of 400 nm with a Tecan Infinite 200 Pro spectrophotometer (Tecan, Switzerland).

The concentration of the phenol groups was determined by the Folin–Ciocalteau (FC) method previously described by Blainski et al. [18]. In addition, 60 µL of FC-reagent were mixed with 20 µL of lignin solution and incubated for 5 min at 21 °C. 200 µL mQH_2_O and 120 µL of a 20% w·v^−1^ sodium carbonate solution were added and the mixture was stirred for 2 h at 800 rpm. Absorbance was then measured spectrophotometrically at 760 nm. The phenol concentration was calculated based on a calibration curve with vanillin as standard.

### 2.7. Size Exclusion Chromatography

Size exclusion chromatography was carried out at 40 °C with an Agilent 1290 Infinity HPLC system (Agilent Technologies, Santa Clara, CA, USA) equipped with an Agilent PL aquagel-OH mixed-H column (8 µm, 7.5 × 300 mm^2^) and an Agilent aquagel-OH Guard column (8 µm) (Agilent Technologies). In addition, 50 mM sodium nitrate (with 0.8 mM sodium azide) with a flow rate of 0.3 mL·min^−1^ constituted the mobile phase. The injection volume was of 50 µL. An Agilent 1260 Infinity refracting index detector (Agilent Technologies, Santa Clara, CA, USA) was used for detection and the molecular weights were determined using poly(sytrenesulfonic acid) sodium salts with a molecular weight ranging from 206 to 1,188,400 Da with a Cirrus Addon (B04.03) for a Chemstation 32 B.04.03[108] software from Agilent Technologies (Santa Clara, CA, USA).

## 3. Results and Discussion

### 3.1. Laccase Immobilization

**Figure 1:**
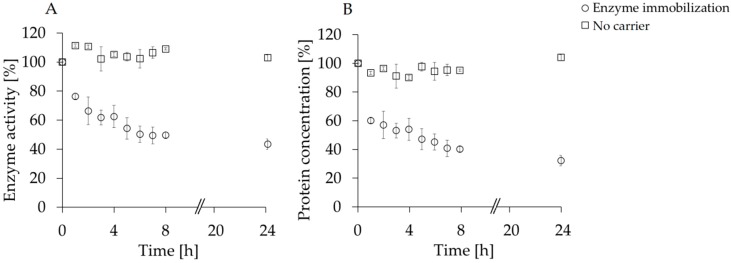
Enzyme activity (**A**) and protein concentration (**B**) analysis (see Section 2.2 and Section 2.3 for assay details) of the supernatant during immobilization of laccase onto PP (poly propylene) compared to a control experiment in the absence of the carrier. All experiments were performed in duplicates.

Starting from previous reports on the possibility to load Accurel MP 1000 beads (PP beads) with various hydrolytic enzymes. In this study, laccase from *Myceliophthora thermophila* was adsorbed on the same polypropylene-based carrier. As reported in Figure 1, according to enzymatic activity analysis of the supernatant, around 50% of the enzyme was bound to the PP beads after 8 h of reaction, while after 24 h the percentage increased to 57% (Figure 1A). These data are confirmed by the protein concentration analysis (Figure 1B) that shows a similar trend. Blanks consisting only of the MtL solution in NaPi buffer showed no significant activity or concentration changes over time. Any attempt of immobilizing higher amounts of MtL onto the PP beads led to lower immobilization yields and less active preparations. These results are in line with what previously reported by de Oliveira et al., which demonstrated that, for the immobilization of *Yarrowia lipolytica* lipase, a higher initial protein contents have not increased the hydrolytic activity neither the yields probably due to the fact that at this value the carrier Accurel MP1000 had achieved maximum enzyme load and higher protein contents caused carrier overcrowding [19].

Several carriers such as polyamide 6,6 (nylon) [20], poly(vinyl alcohol) [21], SiO_2_ nanocarriers [22], poly(sodium 4-sytrenesulphonate)/poly(allylamine hydrochloride)-coated silanized alumina particles [23], and epoxy functionalized polyethersulfones [12] were previously used for laccase immobilization, while PP beads have not yet been described as laccase carrier. Starting from the work carried out by Misra and co-workers, which reported the covalent immobilization of laccase from *Trametes versicolor* on epoxy activated polyethersulfone beads [12], we tried to covalently immobilize MtL on similar carriers (carrying different spacers) in order to obtain a stable, covalently-bonded enzymatic preparation. Unfortunately, in our case, every attempt of covalently immobilizing the MtL onto epoxy-activated beads led to non-active immobilizates. For this reason, all further experiments were carried out using MtL immobilized on PP beads (MtL–PP), since it resulted in being the most active obtained preparation.

### 3.2. Lignin Polymerization

#### Fluorescence Intensity and Phenol Content

**Figure 2:**
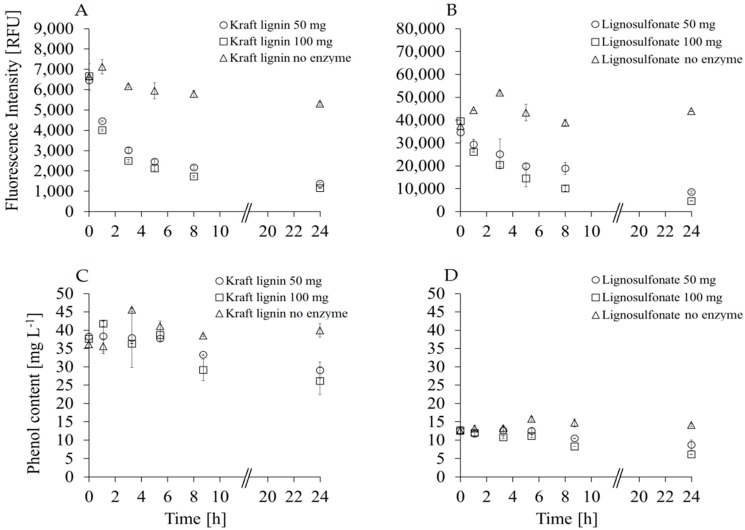
Fluorescence intensities (KL (Kraft Lignin) (**A**) and LS (Lignosulfonate) (**B**)) and phenol content (KL (**C**) and LS (**D**)) of lignins polymerized with 50 and 100 mg of immobilized laccase for 24 h. All experiments were performed in triplicates with the corresponding standard deviation.

Fluorescence intensity and phenol content (see Section 2.6 for assay details) were determined in order to monitor the polymerization process [9]. A 4.6-fold decrease of the fluorescence intensity from 6650 RFU (Relative Fluorescence Units) to 1150 RFU was seen when KL was treated with 100 mg of MtL-PP (0.3% w·w^−1^ MtL with respect to the total amount of lignin) compared to controls with a stable fluorescence intensity over 24 h (Figure 2A, and Table S1 in supporting information). Interestingly, when the KL was treated with 50 mg MtL-PP (corresponding to 0.15% w·w^−1^ MtL with respect to the total amount of lignin), a similar reduction of the fluorescence intensity was observed. Polymerization of lignosulfonates (LS) with 50 and 100 mg MtL–PP also led to comparable results with a fluorescence intensity reduction from 39,565 RFU to 4640 RFU for the 100 mg Mtl-PP sample, corresponding to an 8.5-fold decrease. Results obtained using 50 mg of MtL-PP showed the same trend in the decrease, but led to a higher RFU value at 24 h (8528 RFU, Figure 2B, Table S1). Differences in fluorescence intensity observed for the two lignins could be due to the different wood origin and also due to pretreatment processes [19]. The fluorescence of lignin is originated from several groups that are present in the lignin like phenylcoumaron, stilbene, carbonyl, etc. [24]. Disrupting the lignin structure (in our case due to polymerization reaction) leads to a reduction of the fluorescence intensity [5,8]. During the oxidative reaction of lignin with the immobilized laccase C–C, aryl–aryl, ether, or aryl–alkyl linkages are created [25]. In addition, the laccase from *Myceliophthora thermophila* has a low redox potential, and, in combination with the absence of natural or synthetic mediators, the polymerization of the non-phenolic parts of the lignin cannot be expected [26,27,28].

The phenol content showed a similar trend like the fluorescence intensity during enzymatic polymerization of the lignins. In this case, the decrease was 1.5-fold for the KL and 2.3-fold for LS when 100 mg MtL-PP were used (Figure 2D, Table S2 in supporting information). The results for 50 mg MtL-PP showed a far smaller decrease of the phenol content for both lignins, indicating that the amount of MtL loaded on the PP beads is important for the polymerization process. With the same amount of immobilized laccase applied, a lower content of phenolic hydroxyl groups was measured for LS when compared to KL (Figure 2C, Table S2). A rational explanation could be derived from the fact that KL has a lower starting *M*_w_ than LS [3]. The fragmentation of the lignin macromolecules arises through cleaving of linkages holding the phenylpropane units together with a further formation of free phenolic hydroxyl groups [29]. Hence, cleavage of the α-aryl and β-aryl ether bonds in phenolic lignin units and β-aryl ether bonds in non-phenolic lignin units [29,30]. The α-aryl ether linkages in the phenolic units of the KL are cleaved and quinone methide intermediates are generated. In the non-phenolic units, the β-aryl ether linkages are cleaved by ionized hydroxyl groups present on the α- and γ-carbon [29]. From these data, we can conclude that a modification of the KL and LS occurred after MtL-PP treatment in various reaction conditions. Hence, SEC (Size Exclusion Chromatography) analysis will confirm the detailed change during the oxidation process of the catalyst.

### 3.3. Oxygen Consumption during Lignin Polymerization

**Figure 3:**
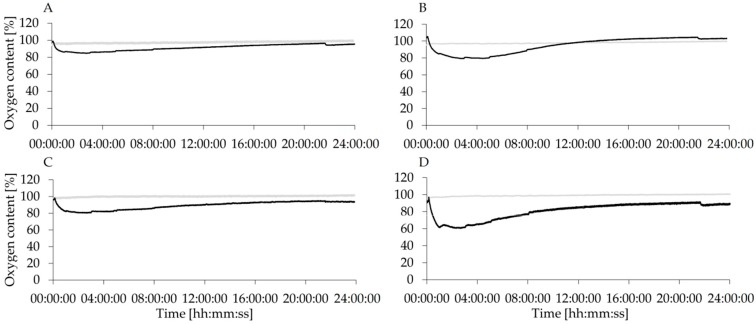
Consumption of oxygen during polymerization of lignin with immobilized laccase (MtL–PP). The lignins were polymerized with 50 mg (LS: **A**, KL: **C**) and 100 mg (LS: **B**, KL: **D**) MtL–PP, respectively. The grey line represents the blank samples (no biocatalyst addition).

The Firesting device, previously used by Greimel et al. to detect the oxidation of fatty acids in alkyd resins [17], was used to monitor the oxygen consumption during the oxidation process of lignin. MtL–PP oxidation of LS (Figure 3A) led to a drop of the oxygen content to around 80% for 100 mg and around 85% for 50 mg MtL–PP beads. During the reaction time, the oxygen content was rising again until it nearly reached the initial value of around 100%. This indicates the capacity of laccase to form radicals during the oxidation process of the lignins, and, therefore, further polymerization is possible. Figure 3 is also showing that laccase is consuming the oxygen during the oxidation of the substrate [31]. The more oxygen that was consumed, the higher the oxidation potential of the catalyst should be, which, for example, is demonstrated for LS with the higher Mtl-PP amount that also results in a more pronounced change of the fluorescence intensity and phenol content and as later reported also on the molecular weight (SEC results).

The same trend was also seen with KL (Figure 3C,D) for both amounts of MtL–PP applied. The oxygen content decreased to around 80% for 50 mg MtL–PP beads and to around 60% for 100 mg. Here, the same trend as for the LS was exhibited. Additionally, the oxygen content is also rising over the reaction time of 24 h almost back to the initial value. The declining oxygen content during laccase mediated reaction with laccases from *Trametes hirsuta*, *Trametes villosa*, and *Myceliophthora thermophila* [8,9] was previously reported in the case of lignin polymerization using free enzymes. In our study, the oxygen content rose again after more than 8 h for both lignins, indicating that the polymerization was lasting longer than 8 h which is also emphasized by the SEC results due to a sharp increase of the *M*_w_ between 8 and 24 h (see Section 3.4). It is also shown that the higher amount of MtL-PP led to a more pronounced decrease of the oxygen consumption during the polymerization process of both lignins.

### 3.4. Molecular Weight Changes during Polymerization

**Figure 4:**
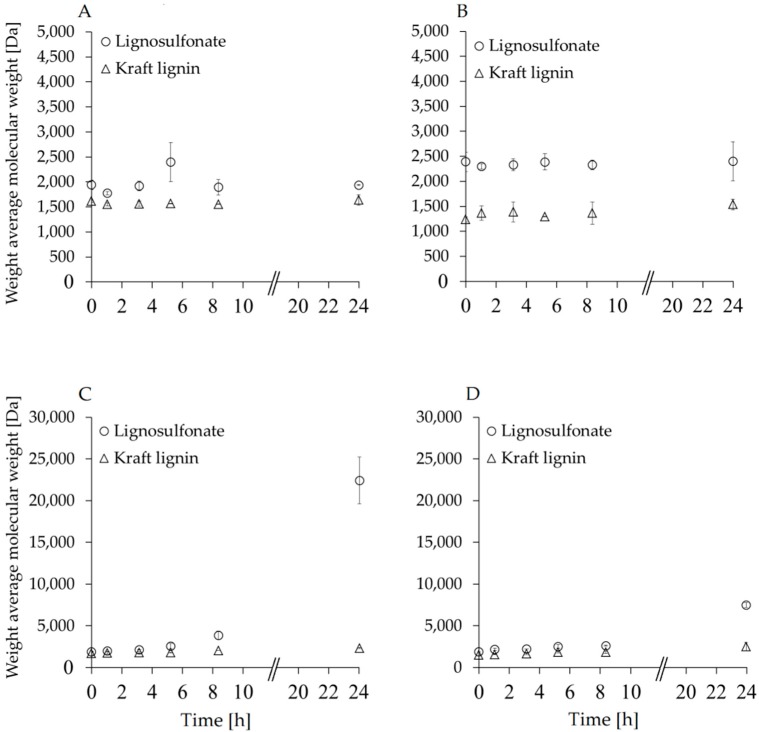
Monitoring of the lignin samples’ stability over the reaction period. Weight average molecular weights (*M*_wS_) of KL and LS without (**A**) and with (**B**) PP beads (without biocatalyst) are presented. LS and KL with 100 mg (**C**) and 50 mg (**D**) MTL–PP beads over 24 h of polymerization are shown. All experiments were performed in duplicates with the corresponding standard deviation.

Since the determination of the weight average molecular weight (*M*_w_) is the method of choice to determine the polymerization degree, SEC analyses were performed. Lignin samples were incubated with and without PP beads for 24 h to demonstrate that the immobilized MtL is necessary for the successful polymerization. Figure 4A shows the 24 h time course reaction in the absence of PP beads. Figure 4B shows the same reaction when unloaded PP beads were added to the lignin solution. The data show no change in the *M*_w__s_ over time, as expected in absence of the biocatalyst. Detailed data are presented in Table S3 including the polydispersity of the polymerized samples that is important for further applications.

The *M*_w_ results of polymerized lignin samples are presented in Figure 4C,D. 50 mg (Figure 4C) and 100 mg (Figure 4D) MtL–PP beads were tested as reported for the other analysis. A 12.0-fold increase of the *M*_w_ was measured for enzymatic polymerization of LS after 24 h when 100 mg beads were used, whereas, for KL, only a 1.4-fold increase was seen. This corresponds to the *M*_w_ of 22,400 Da and 2300 Da for the LS and the KL, respectively. During the reaction of laccase mediated oxidation of lignins, C–C, C–O bonds are also formed to form dimers, which can then lead to the formation of oligomers or polymers [7]. Increasing the polymerization process is possible via addition of pure oxygen to the reaction mixture [8]. When 50 mg MtL–PP were used, a 4.0-fold and 1.7-fold *M*_w_ increase was detected for LS and KL, respectively (Figure 4C), corresponding to 7500 Da and 2500 Da. Expectedly, no changes in *M*_w_ was seen when the lignins were incubated with carrier alone (without immobilized laccase). Without addition of pure oxygen supply, a much lower increase of the *M*_w_ was seen, in agreement to our previous studies with free laccases [8]. The results clearly demonstrate that the amount of used biocatalyst is strongly influencing the polymerization process. In fact, using a larger amount of catalysts frequently results in higher conversion rates and yields. The fact that a higher amount of enzyme leads to a higher amount of metabolites was also published by Crestini et al. in 1998 [32]. The SEC data are in line with the Firesting’s results, which show how the oxygen content is decreasing in a more pronounced way when a higher amount of MtL-PP was used. The *M*_w_ spectra after SEC analysis are presented with Figures S1 and S2 for 100 and 50 mg MtL-PP in the supplementary information.

The polymerization of LS was more efficient when a higher amount of MtL–PP with respect to the total amount of starting lignin was used. On the other hand, no great differences were observed for the polymerization of KL. In conclusion, immobilized MtL on PP beads seems a promising starting point for further developments of laccase immobilization techniques, since it reported being very active in the polymerization of various industrial lignins. For example, Misra et al. [12] reported an improved laccase activity when immobilized vs. the free enzyme, but we are aware that the immobilization of laccases on a carrier can also lead to a loss of enzyme activity as reported by other groups [2].

## 4. Conclusions

A laccase from *Myceliophthora thermophila* was successfully immobilized on Accurel MP1000 beads with a binding efficiency of 67.7% within 24 h. Kraft lignin and lignosulfonate polymerizations were successfully performed using various amounts of the immobilized enzyme. The higher amount of the beads (100 mg) led to more pronounced polymerization of LS leading to a 12.0-fold increase in the obtained *M*_w_, while, for KS, no big differences were detected when 50 or 100 mg of beads were used (1.7 and 1.4-fold *M*_w_ increase, respectively). This work forms the basis for further applications of immobilized laccases for biotransformation of polymeric substrates, allowing recycling of the enzymes to develop economic and environmental friendly processes [33].

## Supplementary Materials

The following are available online at www.mdpi.com/2073-4360/8/8/280/s1, with Table S1: Fluorescence intensity of MTL–PP polymerized KL and lignosulfonate over 24 h of polymerization. The data are presented in RFU with the corresponding standard deviation of triplicates; Table S2: Phenol content of MTL–PP polymerized lignins from 0 to 24 h polymerization. The data were analyzed in triplicates and the phenol content is presented in mg·L^−1^ with the standard deviation; Table S3: Size exclusion chromatography results of LS and KL untreated and treated with 50 and 100 mg Mtl-PP. The weight-average molecular weight *M*_w_ [Da], the number-average molecular weight *M*_n_ [Da] and the polydispersity PD at the beginning of the reaction and after 24 h is presented; Figure S1: *M*_w_ spectra of (A) KL and (B) LS for 100 mg MtL–PP., Curve 1 (red) demonstrates the polymerization without MTL-PP and curve 2 (grey) the polymerization after 24 h of polymerization; Figure S2: *M*_w_ spectra of (A) KL and (B) for 50 mg MtL–PP. LS. Curve 1 (black) demonstrates the polymerization without MTL-PP and curve 2 (grey) the polymerization after 24 h of polymerization.

## Abbreviations

MTL	*Myceliophthora thermophila* laccase
PP	polypropylene
RFU	relative fluorescence units
SEC	size exclusion chromatography
FC	Folin–Cioulteau
NaPi	sodium phosphate
BCA	bicinchoninic acid
kat	katal
ABTS	2,2′-Azino-di-(3-ethylbenzthiazolin-6-sulfonsäure)
KL	Kraft lignin
LS	lignosulfonate
MtL–PP	*Myceliophthora thermophila* laccase immobilized on polypropylene beads

## References

[1] Azadi P., Inderwildi O.R., Farnood R., King D.A. (2013). Liquid fuels, hydrogen and chemicals from lignin: A critical review. Renew. Sustain. Energy Rev..

[2] Crestini C., Crucianelli M., Orlandi M., Saladino R. (2010). Oxidative strategies in lignin chemistry: A new environmental friendly approach for the functionalisation of lignin and lignocellulosic fibers. Catal. Today.

[3] Areskogh D., Li J., Gellerstedt G., Henriksson G. (2010). Investigation of the Molecular Weight Increase of Commercial Lignosulfonates by Laccase Catalysis. Biomacromolecules.

[4] Yousuf M., Mollah A., Palta P., Hess T.R., Vempati R.K., Cocke D.L. (1995). Chemical and physical effects of sodium lignosulfonate superplasticizer on the hydration of portland cement and solidification/stabilization consequences. Cem. Concr. Res..

[5] Nugroho Prasetyo E., Kudanga T., Østergaard L., Rencoret J., Gutiérrez A., del Río J.C., Ignacio Santos J., Nieto L., Jiménez-Barbero J., Martínez A.T. (2010). Polymerization of lignosulfonates by the laccase-HBT (1-hydroxybenzotriazole) system improves dispersibility. Bioresour. Technol..

[6] Gouveia S., Fernández-Costas C., Sanromán M.A., Moldes D. (2012). Enzymatic polymerisation and effect of fractionation of dissolved lignin from Eucalyptus globulus Kraft liquor. Bioresour. Technol..

[7] Riva S. (2006). Laccases: Blue enzymes for green chemistry. Trends Biotechnol..

[8] Ortner A., Huber D., Haske-Cornelius O., Weber H.K., Hofer K., Bauer W., Nyanhongo G.S., Guebitz G.M. (2015). Laccase mediated oxidation of industrial lignins: Is oxygen limiting?. Process Biochem..

[9] Huber D., Ortner A., Daxbacher A., Nyanhongo G.S., Bauer W., Guebitz G.M. (2016). Influence of oxygen and mediators on laccase catalyzed polymerization of lignosulfonate. ACS Sustain. Chem. Eng..

[10] Fiţigău I.F., Peter F., Boeriu C.G. (2013). Oxidative polymerization of lignins by laccase in water-acetone mixture. Acta Biochim. Pol..

[11] Durán N., Rosa M.A., D’Annibale A., Gianfreda L. (2002). Applications of laccases and tyrosinases (phenoloxidases) immobilized on different supports: A review. Enzym. Microb. Technol..

[12] Misra N., Kumar V., Goel N.K., Varshney L. (2014). Laccase immobilization on radiation synthesized epoxy functionalized polyethersulfone beads and their application for degradation of acid dye. Polymer.

[13] Hublik G., Schinner F. (2000). Characterization and immobilization of the laccase from Pleurotus ostreatus and its use for the continuous elimination of phenolic pollutants. Enzym. Microb. Technol..

[14] Cantone S., Ferrario V., Corici L., Ebert C., Fattor D., Spizzo P., Gardossi L. (2013). Efficient immobilisation of industrial biocatalysts: Criteria and constraints for the selection of organic polymeric carriers and immobilisation methods. Chem. Soc. Rev..

[15] Hanefeld U., Gardossi L., Magner E. (2009). Understanding enzyme immobilisation. Chem. Soc. Rev..

[16] Pellis A., Ferrario V., Zartl B., Brandauer M., Gamerith C., Herrero Acero E., Ebert C., Gardossi L., Guebitz G.M. (2016). Enlarging the tools for efficient enzymatic polycondensation: Structural and catalytic features of cutinase 1 from *Thermobifida cellulosilytica*. Catal. Sci. Technol..

[17] Greimel K.J., Perz V., Koren K., Feola R., Temel A., Sohar C., Acero E.H., Klimant I., Guebitz G.M. (2013). Banning toxic heavy-metal catalysts from paints: Enzymatic cross-linking of alkyd resins. Green Chem..

[18] Blainski A., Lopes G.C., de Mello J.C.P. (2013). Application and analysis of the folin ciocalteu method for the determination of the total phenolic content from *Limonium brasiliense* L.. Molecules.

[19] Donaldson L.A., Radotic K. (2013). Fluorescence lifetime imaging of lignin autofluorescence in normal and compression wood. J. Microsc..

[20] Silva C., Silva C.J., Zille A., Guebitz G.M., Cavaco-Paulo A. (2007). Laccase immobilization on enzymatically functionalized polyamide 6,6 fibres. Enzym. Microb. Technol..

[21] Yinghui D., Qiuling W., Shiyu F. (2002). Laccase stabilization by covalent binding immobilization on activated polyvinyl alcohol carrier. Lett. Appl. Microbiol..

[22] Patel S.K.S., Kalia V.C., Choi J.-H., Haw J.-R., Kim I.-W., Lee J.K. (2014). Immobilization of laccase on SiO nanocarriers improves its stability and reusability. J. Microbiol. Biotechnol..

[23] Crestini C., Perazzini R., Saladino R. (2010). Oxidative functionalisation of lignin by layer-by-layer immobilised laccases and laccase microcapsules. Appl. Catal. A Gen..

[24] Albinsson B., Li S., Lundquist K., Stomberg R. (1999). The origin of lignin fluorescence. J. Mol. Struct..

[25] Kudanga T., Prasetyo E.N., Sipilä J., Guebitz G.M., Nyanhongo G.S. (2010). Reactivity of long chain alkylamines to lignin moieties: Implications on hydrophobicity of lignocellulose materials. J. Biotechnol..

[26] Madhavi V., Lele S.S. (2009). Laccase: Properties and Applications. Bioresources.

[27] Bourbonnais R., Paice M., Reid I., Lanthier P., Yaguchi M. (1995). Lignin oxidation by laccase isozymes from Trametes versicolor and role of the mediator 2,2′-azinobis(3-ethylbenzthiazoline-6-sulfonate) in kraft lignin depolymerization. Appl. Environ. Microbiol..

[28] Berka R., Schneider P., Golightly E., Brown S., Madden M., Brown K., Halkier T., Mondorf K., Xu F. (1997). Characterization of the gene encoding an extracellular laccase of Myceliophthora thermophila and analysis of the recombinant enzyme expressed in Aspergillus oryzae. Appl. Envir. Microbiol..

[29] Chakar F.S., Ragauskas A.J. (2004). Review of current and future softwood kraft lignin process chemistry. Ind. Crops Prod..

[30] Gierer J. (1980). Chemical aspects of kraft pulping. Wood Sci. Technol..

[31] Bento I., Silva C.S., Chen Z., Martins L.O., Lindley P.F., Soares C.M. (2010). Mechanisms underlying dioxygen reduction in laccases. Structural and modelling studies focusing on proton transfer. BMC Struct. Biol..

[32] Crestini C., Argyropoulos D.S. (1998). The early oxidative biodegradation steps of residual kraft lignin models with laccase. Bioorg. Med. Chem..

[33] Pellis A., Herrero Acero E., Ferrario V., Ribitsch D., Guebitz G.M., Gardossi L. (2016). The Closure of the Cycle: Enzymatic Synthesis and Functionalization of Bio-Based Polyesters. Trends Biotechnol..

