# Supplementary material for: Polymerization of Various Lignins via Immobilized Myceliophthora thermophila Laccase (MtL)
Source: Polymers (Basel). 2016 Aug 3;8(8):280. doi: 10.3390/polym8080280 (PMC6431913; doi:10.3390/polym8080280)
Supplement: Supplementary file 1 [file polymers-08-00280-s001.pdf]

Supplementary Materials: Polymerization of Various Lignins via Immobilized *Myceliophthora thermophila* Laccase (MtL)

Daniela Huber, Alessandro Pellis, Andreas Daxbacher, Gibson S. Nyanhongo and Georg M. Guebitz

Table S1. Fluorescence intensity of MTL-PP polymerized KL and lignosulfonate over 24 h of polymerization. The data are presented in RFU with the corresponding standard deviation of triplicates.

Time points	Fluorescence intensity [RFU] of LS			Fluorescence intensity [RFU] of KL		
	50 mg MTL-PP	100 mg MTL-PP	No MTL-PP	50 mg MTL-PP	100 mg MTL-PP	No MTL-PP
0 h	34,521.5 ± 3639.5	39,564.5 ± 17.7	37,392.7 ± 360.0	6448.3 ± 497.5	6653.8 ± 40.7	6649.8 ± 627.7
1 h	29,353.5 ± 2239.4	26,149.8 ± 29.3	44,302.5 ± 215.7	4444.5 ± 33.2	4001.0 ± 45.3	7116.3 ± 348.4
3 h	25,130.7 ± 6583.6	20,514.3 ± 448.1	52,035.7 ± 767.1	3020.5 ± 124.5	2498.8 ± 73.8	6161.0 ± 80.6
5 h	19,902.6 ± 937.7	14,472.7 ± 344.6	43,284.1 ± 3580.0	2457.5 ±119.5	2129.8 ± 129.8	5942.3 ± 399.9
8 h	18,947.0 ± 2682.1	10,096.8 ± 631.8	38,849.8 ± 1239.1	2167.0 ± 93.3	1732.5 ± 47.4	5786.8 ± 172.2
24 h	8528.3 ± 484.7	4640.25 ± 544.8	43,875.7 ± 93.5	1382.0 ± 66.5	1149.4 ± 12.8	5301.0 ± 79.2

Table S2. Phenol content of MTL-PP polymerized lignins from 0 to 24 h polymerization. The data were analyzed in triplicates and the phenol content is presented in mg·L^{-1} with the standard deviation.

Time points	Phenol content [mg·L^{-1}] of LS			Phenol content [mg·L^{-1}] of KL		
	50 mg MTL-PP	100 mg MTL-PP	No MTL-PP	50 mg MTL-PP	100 mg MTL-PP	No MTL-PP
0 h	12.6 ± 0.5	12.7 ± 0.1	12.5 ± 0.3	38.3 ± 3.1	37.7 ± 4.8	36.1 ± 2.4
1 h	11.7 ± 0.7	11.9 ± 0.7	13.1 ± 0.9	38.3 ± 4.2	41.7 ± 0.6	35.6 ± 2.1
3 h	12.6 ± 1.2	10.7 ± 0.4	13.2 ± 0.5	37.9 ± 8.1	36.3 ± 0.1	45.6 ± 0.4
5 h	12.5 ± 1.2	11.1 ± 0.5	15.8 ± 0.6	37.7 ± 0.9	38.7 ± 1.4	41.1 ± 1.5
8 h	10.5 ± 0.1	8.3 ± 0.1	14.7 ± 1.1	33.3 ± 0.2	29.1 ± 2.9	38.5 ± 0.2
24 h	8.7 ± 1.4	6.1 ± 0.1	14.1 ± 0.4	29.0 ± 2.3	26.1 ± 3.7	39.9 ± 1.9

Table S3. Size exclusion chromatography results of LS and KL untreated and treated with 50 and 100 mg Mtl-PP. The weight-average molecular weight M_w [Da], the number-average molecular weight M_n [Da] and the polydispersity PD at the beginning of the reaction and after 24 h is presented.

LS	M_w [Da]		M_n [Da]		PD	
	0 h	24 h	0 h	24 h	0 h	24 h
No PP	1900	1900	800	800	2.4	2.4
100 mg PP	2300	2400	1100	1100	2.1	2.2
50 mg Mtl-PP	1900	7500	800	1500	2.4	4.9
100 mg Mtl-PP	1900	22,400	800	1500	2.4	15.7
**KL**	**M_w [Da]**		**M_n [Da]**		**PD**	
	**0 h**	**24 h**	**0 h**	**24 h**	**0 h**	**24 h**
No PP	1600	1600	900	900	1.8	1.9
100 mg PP	2400	2400	1100	1100	1.7	2.2
50 mg Mtl-PP	1500	2500	800	1500	2.4	5.0
100 mg Mtl-PP	1700	2300	900	1000	1.9	2.4

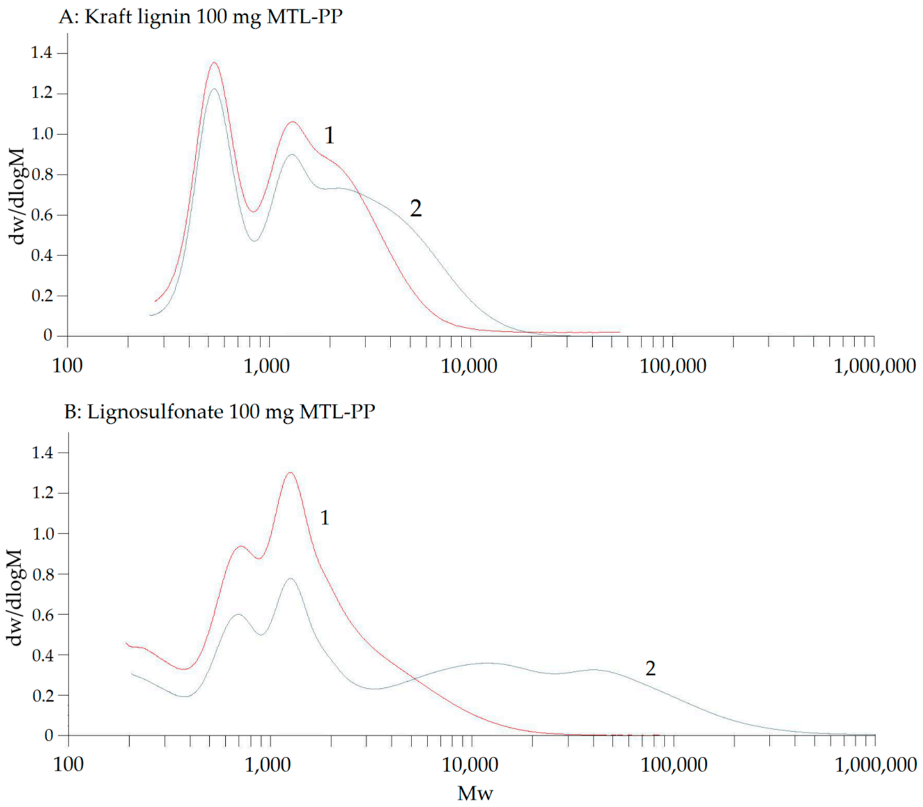

Figure S1. M_w spectra of (**A**) KL and (**B**) LS for 100 mg MtL-PP. Curve 1 (red) demonstrates the polymerization without MTL-PP and curve 2 (grey) the polymerization after 24 h of polymerization.

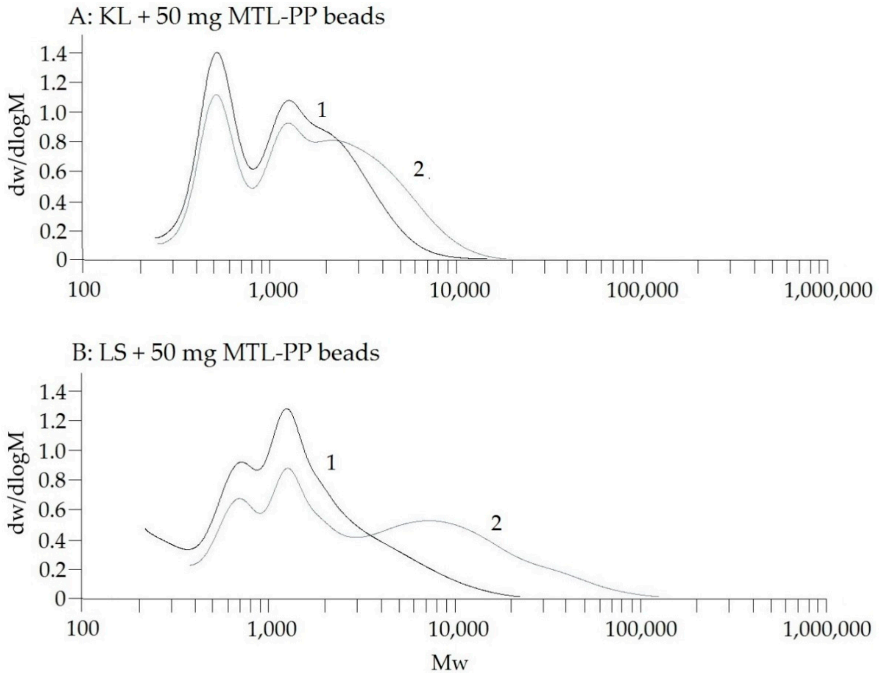

Figure S2. M_w spectra of (**A**) KL and (**B**) for 50 mg MtL-PP. LS. Curve 1 (black) demonstrates the polymerization without MTL-PP and curve 2 (grey) the polymerization after 24 h of polymerization.

© 2016 by the authors; licensee MDPI, Basel, Switzerland. This article is an open access article distributed under the terms and conditions of the Creative Commons Attribution (CC-BY) license (http://creativecommons.org/licenses/by/4.0/).
